# Restore cervical sagittal alignment by cervical disc arthroplasty and systematic total bilateral uncuscectomy in severe spondylosis: A prospective study

**DOI:** 10.1016/j.bas.2023.101765

**Published:** 2023-06-12

**Authors:** Henri-Benjamin Pouleau, Olivier De Witte, Benjamin Dhaene, Alexandre Jodaïtis

**Affiliations:** a University Hospital Center Tivoli, La Louvière, Department of Neurosurgery, Belgium; b Academic Hospital Center Erasme, Bruxelles, Department of Neurosurgery, Belgium; c Academic Hospital Center Erasme, Bruxelles, Chief of Department of Neurosurgery, Belgium; d University Hospital Center Tivoli, La Louvière, Chief of Department of Radiology, Belgium; e University Hospital Center Tivoli, La Louvière, Chief of Department of Neurosurgery, Belgium

**Keywords:** Uncuscectomy, Uncinectomy, Cervical sagittal balance, Cervical sagittal alignment, Cervical disc arthroplasty, Severe spondylosis

## Abstract

**Introduction:**

Severe spondylosis is common and represents contraindication to achieve cervical disc arthroplasty (CDA).

**Research question:**

Is it possible to restore cervical sagittal alignment using an adequate prosthetic model and performing systematic bilateral total uncuscectomy (or uncinectomy), even in cases of severe spondylosis ?

**Material and methods:**

We propose a prospective clinical and radiological study comparing the evolution of preoperative and postoperative cervical sagittal balance 1 year after the interposition of a prosthesis with mobile bearing and systematic total uncuscectomy. VAS for brachialgia and cervicalgia, NDI, Odom's criteria, C2–C7 Cobb angle, C2–C7 SVA, T1 slope, C2 slope, C1–C2 Cobb angle, and segmental Cobb angle were analyzed preoperatively and 1 year postoperatively.

**Results:**

73 patients for a total of 129 levels treated were analyzed. Patients showed significant improvements in VASb, VASc, NDI, and Odom's criteria one year after surgery without clinical differences in the severe spondylosis subgroup (41 patients for 77 levels treated). Our results showed an increase in the C2–C7 Cobb angle postoperatively and a better correlation between T1 slope and C2–C7 Cobb angle postoperatively than preoperatively. Postoperative radiological results were similar between the spondylosis and non-spondylosis subgroups. However preoperative C2–C7 Cobb angle and preoperative ROM were lower in the severe spondylosis subgroup.

**Discussion and conclusion:**

This study showed the possibility of restoring cervical sagittal balance by performing cervical disc arthroplasty with systematic uncuscectomy, even in cases of severe spondylosis. Moreover, we propose a simplified mathematical formula to preoperatively evaluate the lack of angulation to restore sagittal cervical alignment.

## Highlights

• Severe spondylosis is common and represents contraindication to achieve cervical disc arthroplasty (CDA).
• Treat severe spondylosis by CDA with systematic total bilateral uncuscectomy can restore cervical sagittal alignment.
• Prosthesis with mobile bearing permits to restore the natural motion of the cervical spine.
• Uncuscectomy allows the cervical spine to regain its original mobility.
• A simplified mathematical formula evaluates preoperatively the lack of angulation to restore sagittal cervical alignment.

## Graphical abstract

Correlation showed by linear regression between 1 year postoperative C2C7 Cobb angle and T1 slope in all patients treated by CDA with systematic uncuscectomyModel equation: C2C7 Cobb angle ​= ​−9,8272 ​+ ​0,8064 × T1 slopeR^2^ ​= ​0,574; R ​= ​0,757.

**Figure:**
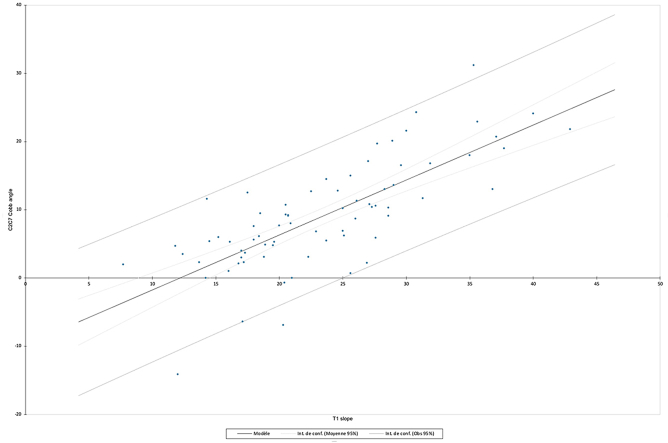

## 1 Introduction

The anterior cervical discectomy and fusion (ACDF) for single-level disc disease was first described by Smith, Cloward, and Robinson in 1958 (Cloward, 1958). This technique is used to relieve mechanical pressure on the spinal nerve roots or spinal cord associated with symptoms refractory to nonsurgical treatment. Typical symptoms include radicular pain, weakness, numbness, and difficulty walking (O'Brien et al., 2019). Cervical radiculopathy or myelopathy can be secondary to disc herniation, anterior osteophyte complexes, or bony spurs that cause spinal canal narrowing, spinal cord compression, or nerve root impingement (Bohlman et al., 1993). It may be successfully used in patients with both single-and multilevel cervical diseases (Bohlman et al., 1993).

Due to concerns regarding the kinematic and biomechanical issues inherent to fusion of the cervical motion segment, investigators have developed cervical disc arthroplasty (CDA). Maintenance of normal spinal kinematics is a primary goal of CDA. The cervical spine is inherently dynamic, with flexion, extension, and lateral bending in addition to anterior and posterior translation (O'Brien et al., 2019). The first artificial cervical disc replacement was a ball-and-socket design (Cummins et al., 1998). Rousseau et al. (2008) examined intervertebral kinematics after the use of this type of cervical prosthesis and concluded that this design did not fully preserve the natural range of motion or the center of motion between flexion and extension. This may be attributed to the absence of translation when using a constrained prosthesis (Barrey et al., 2009; Sang et al., 2020). Different metal-on-metal implants or implants with two metal plates and a fixed core in ultra high molecular weight polyethylene (UHMWPE) were developed last thirty years but they don't allow translation like a natural disc (O'Brien et al., 2019). More recent prosthesis with mobile core permit restoration of natural motion of the cervical spine. The mobile bearing translates to the inferior endplate, allowing flexion, extension, and lateral bending. Prosthesis with mobile core was the only kind of implant used for CDA in our study.

The principle of sagittal balance of the spine, particularly at the lumbar level, has been studied extensively in recent years. The value of applying the concept of sagittal balance in clinical practice is now recognized in an ever-increasing number of publications (Le Huec et al., 2014; Bourghli et al., 2011; Schwab et al., 2007, 2010; Lazennec et al., 2000; Liu et al., 2013; Roussouly and Nnadi, 2010). Schwab established that the ideal threshold of lumbar lordosis to be reached post-operatively is proportional to the pelvic incidence, such that the difference between the pelvic incidence (PI) and total lumbar lordosis (LL) is equal to ​± ​10° (PI-LL ​= ​±10°) (Blondel et al., 2013). The formula LL ​= ​PI ​± ​10° allows for an easy and quick approach and is currently used routinely in many spine care centers (Schwab et al., 2010).

To date, cervical sagittal balance has been less studied, and, to our knowledge, no mathematical formula has been validated in the context of cervical sagittal balance restoration. The estimation of segmental lordosis to be applied when performing ACDF is left to the surgeon's experience.

We assume that it would be possible to restore cervical sagittal balance by using an adequate prosthetic model and performing a systematic total bilateral uncuscectomy (or uncinectomy) to restore the natural mobility of the cervical spine, even in cases of severe spondylosis (severe spondylosis is defined as bridging osteophytes, a loss of disc height greater than 50%, or absence of motion less than 2° (Cummins et al., 1998; Rousseau et al., 2008), corresponding to the grade IV McAfee classification).

Therefore, we propose a prospective clinical and radiological study comparing the evolution of preoperative and postoperative cervical sagittal balance 1 year after the interposition of a prosthesis with mobile bearing and systematic uncuscectomy. We will also attempt to establish a simple mathematical formula to predict the angulation necessary to restore the sagittal cervical balance.

## 2 Materiel and methods

• Arthroplasty with systematic uncuscectomy - surgical technique (Video 1 and 2) (Pouleau et al., 2023)

The standard Smith-Robinson approach to the anterior cervical spine is used with a horizontal incision in the skinfold. The discectomy is performed under a microscope. We expose the posterior longitudinal ligament and medial part of the two uncus. We systematically mill both whole uncus until a thin layer of bone like eggshell is obtained; therefore, we recommend using a 4 ​mm diamond bur. The thin residual layer is carefully removed using a curette or a hook. The endplates are prepared by gently drilling the surface. The posterior longitudinal ligament is open. The widest and deepest implant must be inserted under radiographic guidance. The prosthesis must be neither too thin nor too thick to avoid migration or facet overloading. When the prosthesis is in place, we recommend confirming perfect positioning (lateral and AP) using 3D imaging.

Supplementary video related to this article can be found at https://doi.org/10.1016/j.bas.2023.101765

The following is/are the supplementary data related to this article.

Multimedia component 1 Multimedia component 1

Multimedia component 2 Multimedia component 2

• Recruitment

After approval by the ethics committee, we prospectively recruited patients who underwent CDA with systematic total bilateral uncuscectomy between first April 2018 and first April 2020.

The inclusion criteria were symptomatic radicular or medullary compression with symptoms refractory to nonsurgical treatment. The exclusion criteria were severe facet joint degeneration at the surgical level, trauma, tumor, infection, allergy to materials, hybrid construction (CDA and ACDF), anterior and posterior approaches, and revision at the surgical level. Patients with severe disc degeneration or spondylosis were not excluded from this study.

**Fig. 1:**
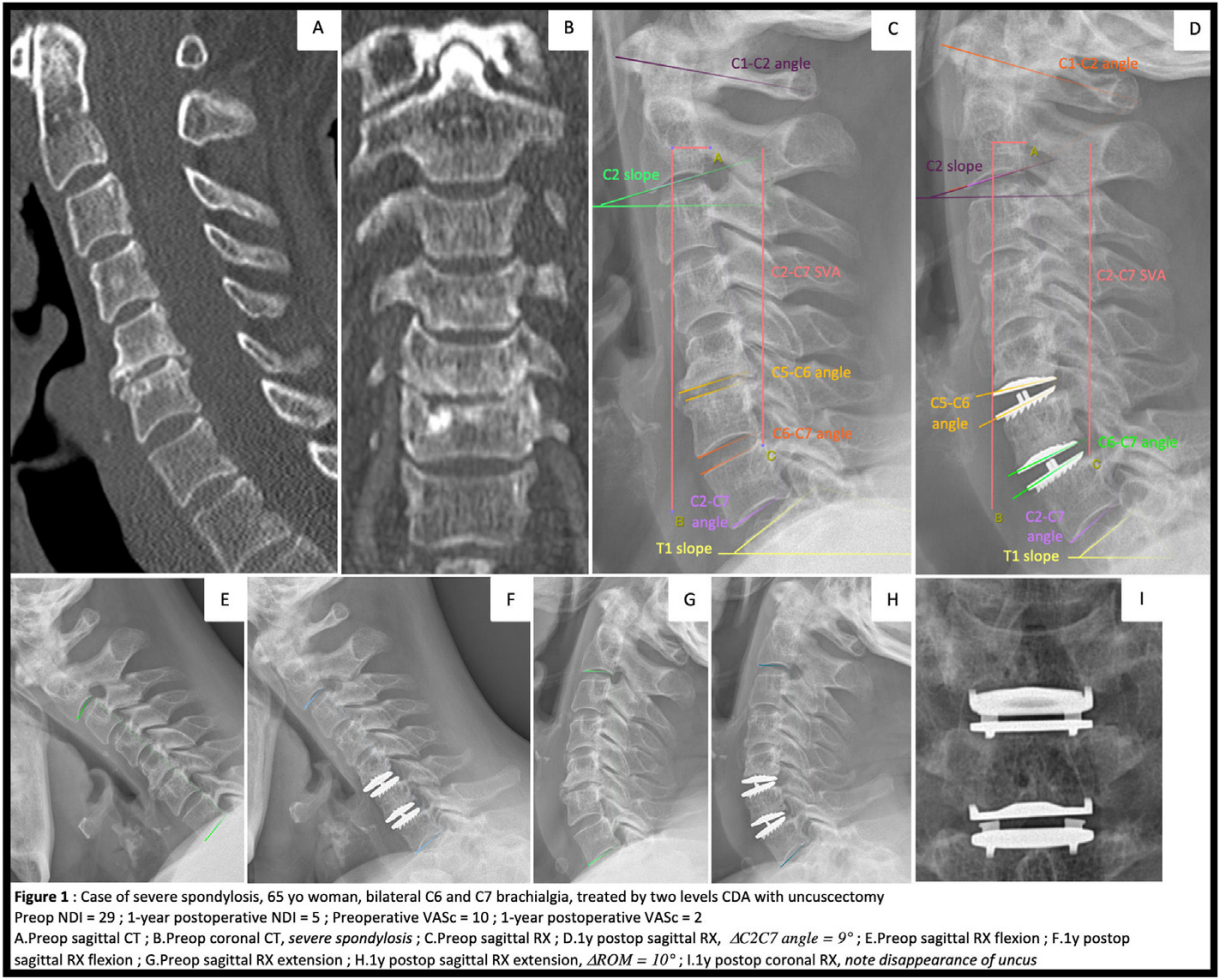
Case of severe spondylosis, 65 yo woman, bilateral C6 and C7 brachialgia, treated by two levels CDA with uncuscectomy Preop NDI ​= ​29; 1-year postoperative NDI ​= ​5; Preoperative VASc ​= ​10; 1-year postoperative VASc ​= ​2 A. Preop sagittal CT; B. Preop coronal CT, severe spondylosis; C. Preop sagittal RX; D.1 ​y postop sagittal RX, *ΔC2C7 angle* ​= ​*9°*; E. Preop sagittal RX flexion; F.1 ​y postop sagittal RX flexion; G. Preop sagittal RX extension; H.1 ​y postop sagittal RX extension, *ΔROM* ​= ​*10°*; 1 ​y postop coronal RX, note disappearance of uncus.

Visual analog scales for brachialgia (VASb), cervicalgia (VASc), and the neck disability index (NDI) were measured before and one year after surgery. Odom's criteria were assessed one year after surgery. Preoperative and 1 year postoperative X-rays were analyzed. C2–C7 Cobb angle (neutral, flexion, and extension), C2–C7 SVA, T1 slope, C2 slope, C1–C2 Cobb angle, segmental Cobb angle at the treated levels and range of motion (ROM) were measured (Fig. 1). Ten cases in our series were re-measured by a radiologist, and no statistically significant difference was found compared to the first examination.

We used Wilk-Shapiro, Mann-Whitney, sign tests, Wilcoxon signed-rank tests, and linear regressions for statistical analyses.

## 3 Results

73 patients meeting the inclusion and exclusion criteria underwent the CDA procedure with systematic bilateral total uncuscectomy and agreed to participate in the study. The average age at the time of surgery was 50,6 (±9,6) years. The male-to-female ratio was similar (36/37). 26 patients received one level of CDA, 38 patients received two levels of CDA, and nine patients received three levels of CDA, for a total of 129 levels.

The median preoperative VASb was 8 [7; 9] and decreased to 0 [0; 3] (p ​< ​0,0001) one year after surgery. The median preoperative VASc was 8 [7; 9] and decreased to 1 [0; 3] (p ​< ​0,0001) one year after surgery. The median preoperative NDI was 28,0 [23; 35], which decreased to 3 [0; 6] (p ​< ​0,0001) one year after surgery. Odom's criteria were 1 (Cloward, 1958; O'Brien et al., 2019) (excellent) one year after surgery.

Preoperative C2–C7 Cobb angle was 5,2° [0; 11,9] and increased to 8,7° [4; 13] postoperatively (p ​< ​0,0002). Preoperative C2–C7 SVA was 3,14 [2,1; 4,1] and decreased to 2,8 [2,1; 3,6] postoperatively (p ​< ​0,05). Preoperative C1–C2 Cobb angle was 31,3° [27; 35,4] and decreased to 28,2° [23,8; 32,6] postoperatively (p ​< ​0,0001). Preoperative C2 slope was 17,4° [13,8; 23] and decreased to 14,7° [11,8; 17] postoperatively (p ​< ​0,0001). Preoperative cervical range of motion was 41° [31,1; 50] and increased to 49,7° [36,4; 58,4] postoperatively (p ​< ​0,001).

In our series, the T1 slope was 22,5° [18; 27,7]. This remained stable pre- and postoperatively.

We found a correlation between the preoperative C2–C7 Cobb and T1 slope (R ​= ​0,486). This correlation increased postoperatively (R ​= ​0,757). The correlation between the C2–C7 SVA and T1 slope increased postoperatively (preoperative R ​= ​0,202; postoperative R ​= ​0,385). We found a correlation between the preoperative C2 slope and preoperative C2–C7 Cobb angle (R ​= ​−0,501) and a similar correlation between the postoperative C2 slope and postoperative C2–C7 Cobb angle (R ​= ​−0,489).

Linear regression between postoperative C2–C7 Cobb and T1 slope allowed us to obtain the following equation: Postoperative C2–C7 Cobb ​= ​−9827 ​+ ​0,806 ​× ​T1 slope (Graph 1).

No major intraoperative complications, particularly damage to the vertebral artery or dural tears, occurred. One patient developed postoperative C5 paresis and recovered completely within a few weeks. No material failure occurred during the one-year follow-up period.

Severe spondylosis subgroup versus no spondylosis (Table 1).

**Table 1:** Severe spondylosis subgroup versus no spondylosis.

	Severe spondylosis subgroup (41)	No severe spondylosis subgroup (32)
Age	53,9 yo (±9,2)	46,3 yo (±8,5)

Postoperative VASb	0 [0; 3]	0 [0; 3]
	*p NS*	
Postoperative VASc	2 [0; 3]	1 [0; 4]
	*p NS*	
Postoperative NDI	3 [1; 6]	1,5 [0; 6]
	*p NS*	
Δ C2–C7 angle	4,4 [-0,5; 12,3]	0,8 [-1,7; 5,1]
	*p* ​< ​*0,01*	
Δ ROM	7,7 [-2,2; 24,9]	1,7 [-7,2; 16,8]
	*p* ​= ​*0,03*	

We compared patients with at least one level of severe spondylosis (bridging osteophytes, loss of disc height greater than 50%, or absence of motion less than 2°, equivalent to grade IV of the McAfee classification) to other patients.

41 patients with severe spondylosis were treated. They were older than other patients: 53,9 yo (±9,2) vs 46,3 yo (±8,5).

No statistical difference was found depending on pre-existing spondylosis about postoperative VASb, VASc, NDI and Odom's criteria.

No statistical difference was found in the postoperative C2–C7 Cobb angle and postoperative C2–C7 SVA between the groups. However, ΔC2-C7 Cobb angle was higher for severe spondylosis subgroup (4,4° [−0,6; 12,3]) than for no spondylosis subgroup (0,8° [−1,7; 5,1]) with statically significative results (p ​< ​0,01). So preoperative C2–C7 Cobb angle was lower in the severe spondylosis subgroup than no spondylosis subgroup. This indicates that the spondylosis subgroup was more unbalanced preoperatively.

Difference between ΔC2-C7 SVA in both groups was not sufficient to be statistically significative.

No statistical difference was found in the postoperative range of motion between the groups. However, ΔROM was significantly higher in the severe spondylosis subgroup (7,7° [−2,2; 24,9]) than in the no spondylosis subgroup (1,7° [−7,2; 16,8]) (p ​= ​0,03). Therefore, the preoperative ROM was lower in the severe spondylosis subgroup than in the no spondylosis subgroup. This indicates that the spondylosis subgroup was less mobile before surgery.

Moreover, the preoperative C1–C2 Cobb angle was 32° [30,3; 35,7] and decreased to 29,7° [25,3; 33,3] postoperatively (p ​< ​0,001) in the severe spondylosis subgroup, and no statistically significant improvement was found in the no spondylosis subgroup. The preoperative C2 slope was 17,3° [15; 24,4] and decreased to 14,9° [12; 17,2] postoperatively (p ​< ​0,001) in the severe spondylosis subgroup, and no statistically significant improvement was found in the no spondylosis subgroup.

### 3.1 Levels subgroups

We noted in our serie that more levels we had to operate, older the patient was:1 level 47 yo (±9,5), 2 levels 51,7 yo (±9,9) and 3 levels 55,3 (±5,5). However, subgroups based on levels and subgroups based on pre-existing severe spondylosis were not the same subgroups: we found for one level subgroup 12 pre-existing spondylosis (14 no spondylosis), for two levels subgroup 22 pre-existing spondylosis (16 no spondylosis) and for three levels 7 pre-existing spondylosis (2 no spondylosis). Of course, the more levels we had to operate on, the older the patient was, and the higher the risk of having at least one level with severe spondylosis; however, severe pre-existing spondylosis represented 46% of one level and 58% of two levels.

No statistical difference was found in the number of treated levels according to postoperative VASb, VASc, NDI, and Odom's criteria.

No statistical difference was found in the postoperative C2–C7 Cobb angle and postoperative C2–C7 SVA depending to the number of treated levels.

No statistical difference was found for ΔC2-C7 Cobb angle and ΔC2-C7 SVA depending on the number of treated levels.

This is probably explained by the fact that an operated disc, owing to neurological compression, does not necessarily lose mobility, allowing the maintenance of sagittal balance. This was confirmed by the absence of statistical differences in postoperative ROM and ΔROM according to the number of treated levels.

## 4 Discussion

Restoration of normal kinematics of the spine is one of the primary goals of CDA. Several meta-analyses have been carried out in recent years and have attempted to prove the superiority of CDA over ACDF, in particular, to avoid adjacent segment degeneration (ASD) (Luo et al., 2018; Lian et al., 2017; Shriver et al., 2016; Zhang et al., 2020; Wang et al., 2020; Latka et al., 2019).

Pseudoarthrosis is another concern associated with ACDF and becomes more prevalent as the number of fused segments increases. Pseudoarthrosis has been reported in 11% of single-level fusions and 27% of multilevel fusions (Bohlman et al., 1993).

Because of these concerns and the desire to preserve motion and return patients to routine activities, the CDA appeared approximately 30 years ago, and many different materials were developed. Every cervical prosthesis has a different conception and, therefore, exhibits a different behavior in the cervical spine. This partly explains the difficulty in drawing reliable conclusions from literature on CDA. However, several meta-analyses have already shown the superiority of CDA over ACDF in terms of pain improvement (Kan et al., 2016; Ma et al., 2016; Zou et al., 2016). However, classical contraindications, particularly severe spondylosis, have limited the use of cervical prostheses.

In our study, patients treated with CDA and systematic total bilateral uncuscectomy showed statistically significant improvements in VASb, VASc, NDI, and Odom's criteria one year after surgery. No clinical difference was found between the severe and non-severe spondylosis subgroups treated with CDA and uncuscectomy. However, severe spondylosis reduces the mobility of the disc and thus its capacity to maintain cervical sagittal alignment. We believe that two points are essential for cervical arthroplasty. First, it involves the use of a prosthesis with the capacity to restore the natural motion of the cervical spine. Second, allow the cervical spine to regain its original mobility. This second step is possible because of the systematic total bilateral uncuscectomy, provided that the facet joints are in good condition. In our study, the sagittal balance was the same postoperatively in the severe spondylosis and non-spondylosis subgroups. However, the correction of sagittal balance postoperatively was more important in the severe spondylosis subgroup. This study argues that severe spondylosis should not be a contraindication for CDA if the original mobility is restored by systematic total bilateral uncuscectomy. We believe that restoring the mobility capacity of the cervical spine and using a prosthesis that mimics the motion of a natural disc will allow the prosthesis to balance itself according to the natural balance of the cervical spine.

However, setting up a prosthesis is not mandatory to restore correct sagittal balance in a neutral position. In this case, we believe that better results can be obtained if the segmental angulation necessary for application during ACDF can be predicted. Our results showed an increase in the C2–C7 Cobb angle postoperatively and a better correlation between the T1 slope and the C2–C7 Cobb angle postoperatively than preoperatively. Moreover, we found a stable correlation between the C2 slope and C2–C7 Cobb angle pre- and postoperatively. Finally, C2 slope decreased postoperatively. Therefore, CDA with a prosthesis mimicking the motion of a natural disc and systematic total bilateral uncuscectomy permit the adaptation of the C2–C7 Cobb angle to the T1 slope, to have a better C2 slope, to increase ROM, and to restore cervical sagittal balance. After performing linear regression, we suggest using this simplified mathematical formula: Postoperative C2–C7 Cobb = (80% T1 slope) – 10°.

Uncuscectomy in the context of CDA is not a common technique reported in literature. M. Makhni shares tips and tricks about CDA (Makhni et al., 2019) and seems to share the same interest in uncuscectomy to prevent the reappearance of osteophytes. Long-term studies will allow us to determine whether uncuscectomy can delay reduction in prosthesis mobility.

No major intraoperative complications or damage to the vertebral arteries was observed. We believe that adequate milling of the uncus with respect to anatomical markers allows for completely safe intervention.

No material failure occurred during the one-year follow-up period. However, long-term studies are necessary to verify the stability of this material, particularly when performing uncuscectomy.

## 5 Conclusions

To our knowledge, this study is the first one in the literature to show the possibility of restoring cervical sagittal balance by performing cervical disc arthroplasty with systematic total bilateral uncuscectomy even in cases of severe spondylosis. Our prospective clinical and radiological results provide interest in a specific surgical technique to reduce cervical pain and improve function one year after surgery. Moreover, we propose a simplified mathematical formula to preoperatively evaluate the lack of angulation to restore sagittal cervical alignment. Long-term studies are necessary to determine whether uncuscectomy can delay the reappearance of osteophytes and certify the stability of the material.

## Funding

The authors declare that no funds, grants, or other support were received during the preparation of this manuscript.

## Author contributions

All authors contributed to the study conception and design. Material preparation, data collection and analysis were performed by HB Pouleau. The first draft of the manuscript was written by HB Pouleau.

## Ethics approval

This study was performed in line with the principles of the Declaration of Helsinki. Approval was granted by the Ethics Committee of Erasme University Bruxelles.

EUDRACT reference: B406201835482.

## Consent to participate

Informed consent was obtained from all individual participants included in the study.

## Consent to publish

The authors affirm that human research participants provided informed consent for publication of the images in Fig. 1.

## FDA approved

The device (Mobi-C) that is the subject of this manuscript is not FDA-approved for this indication (severe spondylosis).

## Declaration of competing interest

A.Jodaïtis receives royalties (annual payments) from Zimmer-Biomet (conceptor MobiC).

Others authors have no relevant financial or non-financial interests to disclose.
